# Comparison of the efficacy and patients’ tolerability of Nepafenac and Ketorolac in the treatment of ocular inflammation following cataract surgery: A meta-analysis of randomized controlled trials

**DOI:** 10.1371/journal.pone.0173254

**Published:** 2017-03-02

**Authors:** Xinyu Zhao, Song Xia, Erqian Wang, Youxin Chen

**Affiliations:** Department of Ophthalmology, Peking Union Medical College Hospital, Chinese Academy of Medical Sciences, Beijing, China; Oregon Health and Science University, UNITED STATES

## Abstract

As a new ophthalmic non-steroidal anti-inflammatory drug (NSAID) with prodrug structure, Nepafenac was supposed to have a better efficacy than conventional NSAIDs both in patients’ tolerability and ocular inflammation associated with cataract surgery. However, many current studies reached contradictory conclusions on the superiority of Nepafenac over Ketorolac. The objective of our study is to evaluate the efficacy and patients’ tolerability of Nepafenac and Ketorolac following cataract surgery. To clarify this, we conducted a meta-analysis of randomized controlled trials. Eleven articles were included in this study. The dataset consisted of 1165 patients, including 1175 cataract surgeries. Among them, 574 patients were in the Nepafenac group and 591 in the Ketorolac group. Our analysis indicated that these two drugs were equally effective in controlling post cataract surgery ocular inflammation, reducing macular edema, achieving a better visual ability and maintaining intraoperative mydriasis during cataract surgery. However, Nepafenac was more effective than Ketorolac in reducing the incidence of postoperative conjunctival hyperemia and ocular discomfort. This meta-analysis indicated that topical Nepafenac is superior to Ketorolac in patients’ tolerability following cataract surgery. However, these two drugs are equally desirable in the management of anterior chamber inflammation, visual rehabilitation and intraoperative mydriasis. Given the limitations in our study, more researches with larger sample sizes and focused on more specific indicators such as peak aqueous concentrations of drugs or PEG2 levels are required to reach a firmer conclusion.

## Introduction

With the significant progress in surgical techniques and apparatus such as phacoemulsification, modern cataract surgery has achieved a reduction of physical surgical trauma and a decrease in the release of prostaglandins, which plays the main role in the progress of postoperative ocular inflammation [1, 2]. However, most patients still manifest clinically significant postoperative ocular inflammation after cataract surgery. Uncontrolled intraocular inflammation may disrupt the blood-ocular barrier and cause the entry of inflammatory cells and cytokines into aqueous humor, leading to patient discomfort, delayed recovery, suboptimal visual outcomes and even further complications such as cystoid macular edema (CME), synechiae formation, raised intraocular pressure (IOP), corneal edema, intraoperative miosis, hyperemia, photophobia and so on [2–4]. In this epoch of patients’ high expectations and premium intraocular lenses, not only suboptimal visual outcomes but also postoperative discomfort are unacceptable to most patients.

Topical steroid therapy could effectively control the postoperative ocular inflammation; however, it may also increase IOP, inhibit wound healing and increase the risk of infection. [5, 6] Currently, more cataract surgeons are becoming interested in avoiding steroid use alone, seeking alternative or complementary drugs which are equally effective but have fewer side-effects [5]. Topical non-steroidal anti-inflammatory drugs (NSAIDs) are commonly applied in the management of noninfectious ocular inflammation following ophthalmic surgery, the combination therapy of NSAIDs and steroid have also shown a synergistic effect on ocular inflammation following cataract surgery [2–3,5–7]. The obvious advantages of NSAIDs over corticosteroids include relative stable IOP, lower risk of secondary infections and extra benefit of analgesia [8–9]. These make NSAIDs a promising agent for cataract surgery.

Most NSAIDs are weakly acidic and ionize in the more basic lachrymal fluid, which limits their corneal penetration. If we adjust the pH of the preparations to improve their ability of corneal penetration, the incidence of ocular irritation and discomfort may also increase [2]. For drug manufacturers who wish to develop these agents, the main challenge is achieving the delicate balance between permeability and patients’ tolerability. As a relative new ophthalmic NSAID, Nepafenac is the only one with a prodrug structure, making it a neutral molecule. This unique property allows it to rapidly penetrate the cornea, after which it is converted by intraocular hydrolases to its more active moiety Amfenac [7, 10]. Theoretically, Nepafenac may have a better efficacy than conventional NSAIDs both in patient tolerability and ocular inflammation associated with cataract surgery.

However, the comparison between Nepafenac and Ketorolac is interesting. The latter was always used as a benchmark, as many previous studies have proven the effectiveness of Ketorolac in the management of both pain and inflammation following cataract surgery [1, 11–13]. Despite Nepafenac’s various theoretical advantages, many current studies failed to detect the superiority of Nepafenac over Ketorolac. Many contradictory conclusions were found both on ocular bioavailability and potency of prostaglandin inhibition, which was closely related to the management of ocular inflammation [5, 14–17]. As Nepafenac is significantly more expensive, we need more solid evidences to conclude whether a change in our routine regimen of postoperative therapy from Ketorolac to Nepafenac is appropriate.

Until now, no meta-analysis in this field has focused on this problem. Thus we undertook a meta-analysis to evaluate the efficacy and patient tolerability of Nepafenac and Ketorolac for the prevention and treatment of pain and inflammation following cataract surgery, in order to provide a reference for the decision-making of ophthalmologists.

## Materials and methods

This meta-analysis was performed strictly according to the guidelines, the ‘preferred reporting items for systematic reviews and meta-analysis (the ‘PRISMA’ statement)’ [18].

### Search strategy

The PubMed, Embase, and the Cochrane Central Register of Controlled Trials were searched from their earliest entries through December, 2016. The following keywords or corresponding Medical Subject Headings (Mesh) were used: “Nepafenac”, “Ketorolac”, “non-steroidal anti-inflammatory drugs”, “NSAIDs” and “cataract surgery”. The detailed electronic search strategy of PubMed was (((cataract[Title/Abstract]) OR "Cataract"[Mesh])) AND ((((((((NSAIDs[Title/Abstract]) OR non-steroidal anti-inflammatory drugs[Title/Abstract]) OR non-steroidal anti-inflammatory drug[Title/Abstract]) OR Nepafenac[Title/Abstract]) OR Ketorolac[Title/Abstract]) OR "Anti-Inflammatory Agents, Non-Steroidal"[Mesh]) OR "Ketorolac"[Mesh]) OR "nepafenac" [Supplementary Concept]) Filters: Humans. The searches started at November 15, 2016 and ended at December 1, 2016. The reference lists of the relevant articles were also manually examined to further identify potentially related studies. No language restriction was imposed.

### Inclusion criteria and exclusion criteria

Inclusion criteria were (1)Participants: patients with visually significant cataract; (2)Intervention: cataract surgery; (3)Comparison: postoperative pain and ocular inflammation were managed with the use of Nepafenac versus Ketorolac; (4)Outcomes: at least one of the followings: best corrected visual acuity (BCVA, log MAR scale), anterior chamber inflammation, inflammation free rate, central macular thickness (CMT), intraoperative mydriasis, ocular discomfort, peak drug concentrations and prostaglandin E2 (PEG_2_) levels, conjunctival hyperemia and other complications; (5)Methodological criterion: randomized Controlled Trial.

Exclusion criteria were (1)Other differences between case group and control group beside the administration of Nepafenac and Ketorolac; (2)Insufficient data to estimate a relative risk (RR) or weighted mean difference (WMD); (3)Animal studies or cadaver subjects; (4)Redundant publications.

### Data extraction and assessment of methodological quality

After consecutive procedures of screening titles and abstracts, obtaining the full text of each article and reviewing them, articles that met the eligibility criteria and fail the exclusion criteria were included. Two authors (ZXY and XS) extracted relevant data independently, including the first author’s name, publication year, design, sample size (patients and eyes), group size, average age, gender ratio, application method, other interventional protocols and outcomes. The data of updated studies involving the same cohort of patients were extracted synthetically. The corresponding authors of the included articles would be contacted if the requisite data were unavailable. Using a 12-item scale [19], the methodological quality of each included RCT was assessed by two authors independently; a trial with a score of 7 or more was considered high quality, more than 4 but no more than 7 was considered moderate quality, and no more than 4 was considered low quality. Disagreements were evaluated by kappa text and were resolved by discussing with the corresponding author (CYX).

### Statistical methods

Statistical analyses were performed with StataSE 12.0 software (StataCorp, College Station, TX, USA). The weighted mean difference (WMD) and 95% confidence interval (CI) were calculated for continuous data, and the relative risk (RR) and 95%CI were calculated for dichotomous data. The statistical heterogeneity was tested by Chi-squared test and *I*^*2*^. If heterogeneity was low (*P* > 0.1, *I*^*2*^ < 50%), a fixed-effect model would be used. If heterogeneity was substantial (*P* < 0.1,*I*^*2*^ *>* 50%), both sensitivity analysis and subgroup analyses were performed to identify the source of the heterogeneity. If the heterogeneity could not be eliminated, a random-effect model would be used when the result of meta-analysis had clinical homogeneity, or a descriptive analysis would be used.

Publication bias was assessed by Begg’s funnel plot and the Egger’s linear regression test. For all statistical analyses, with the exception of heterogeneity, a value of *P* < 0.05 was considered to indicate statistical significance.

### Source of funding

No external funding was received in support of this study.

## Results

### Study characteristics

**Fig 1:**
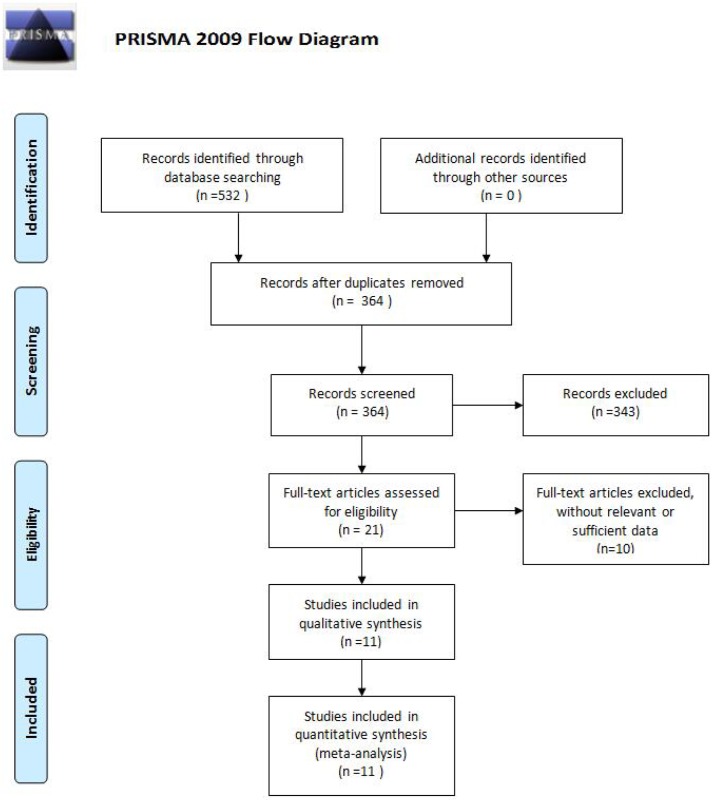
Flowing chart summarizing the selection process.

A total of 532 potentially relevant articles were identified for this meta-analysis. After removing 168 duplicate studies, screening of titles and abstracts and removing 343 unrelated articles, 21 full texts of the left studies were obtained and reviewed. Among them, another 10 studies were excluded for unrelated or insufficient data. Finally, 11 studies were selected for this meta-analysis (Fig 1) [3, 7, 14–17, 20–24]. The dataset consisted of 1165 patients, including 1175 cataract surgeries. Among them, 574 patients were in the Nepafenac group and 591 in the Ketorolac group. The sample sizes of the included studies were between 50 and 200 patients. In each study, the demographic characteristics of the two groups were similar. The main characteristics of the included studies were presented in Table 1 and the literature-exclusion procedures were described in Fig 1. The methodological quality of the included studies was assessed with the 12-item scale (Table 2). The results showed that the average score for the quality of included studies was 10.55±0.93 and all of them were of high quality. There was excellent inter-rater agreement between the investigators regarding eligibility (κ = 0.78).

**Table 1:** Main characteristics of the included studies.

First author	Publication year	Design	Sample size	Eyes	Group size(Patients)	Average age	Gender ratio (Male/female)		Application method		Other intervention protocol	Outcomes
					Nepafenac/Ketorolac	Nepafenac/Ketorolac	Nepafenac	Ketorolac	Nepafenac	Ketorolac		
**Sahu, S**	2015	RCT	64	64	31/33	(60.4±10.2)/(63.4±9.6)	18/13	19/13	Tid for PreOp 1 day and PostOp 6 weeks	Tid for PreOp 1 day and PostOp 6 weeks	Moxifloxacin	Best-corrected visual acuity
											Prednisolone Acetate	Anterior chamber inflammation
**Duong, HV**	2007	Double-masked RCT	178	188	86/92	(69.4±10.6)/(68.9±12.1)	39/47	41/51	Tid for PreOp 3 day and PostOp 1 week	Qid for PreOp 3 day and PostOp 1 week	Gatifloxacin or Moxifloxacin	Best-corrected visual acuity
											Prednisolone Acetate	Anterior chamber inflammation
**Malik, A**	2016	RCT	100	100	50/50	(59.4±8.5)/(58.3±7.8)	24/16	33/17	Tid for PostOp 1 month	Qid for PostOp 1 month	NA	Best-corrected visual acuity
												Anterior chamber inflammation
												Conjunctival hyperemia
**Ramakrishnan, S**	2016	RCT	200	200	100/100	(58.0±8.5)/(59.3±8.7)	NA	NA	Tid for PreOp 1 day and PostOp 4 weeks	Qid for PreOp 1 day and PostOp 4 weeks	Ofloxacin	Best-corrected visual acuity
											Prednisolone Acetate	Central macular thickness
												Intraoperative mydriasis
**Tzelikis, PF**	2015	RCT	86	86	41/45	65.3±7.3	NA	NA	Tid for PreOp 2 day and PostOp 4 weeks	Qid for PreOp 2 day and PostOp 4 weeks	Moxifloxacin	Best-corrected visual acuity
											Prednisolone Acetate	Central macular thickness
**Almeida, DR**	2012	Double-masked RCT	108	108	54/54	72.4±8.2	NA	NA	Qid for PreOp 1 day and PostOp 4 weeks	Qid for PreOp 1 day and PostOp 4 weeks	Gatifloxacin	Best-corrected visual acuity
											Prednisolone Acetate	Total macular volume
												Ocular discomfort
**Nardi, M**	2007	Double-masked RCT	149	149	76/73	77.1/72.9	34/42	28/45	Tid for PreOp 1 day and PostOp 3 weeks	Tid for PreOp 1 day and PostOp 3 weeks	None	Anterior chamber inflammation
												Conjunctival hyperemia
												Ocular discomfort
**Walters, T**	2007	Double-masked RCT	50	50	25/25	(65.9±12.2)/(68.8±10.7)	13/12	13/12	5 times before surgery	5 times before surgery	None	Cmax
**Bucci, FA**	2011	Double-masked RCT	80	80	38/42	NA	NA	NA	5 times before surgery	5 times before surgery	None	Cmax
**Bucci, FA**	2011	Double-masked RCT	80	80	38/42	NA	NA	NA	5 times before surgery	5 times before surgery	None	PEG2
**Zanetti, FR**	2012	RCT	70	70	35/35	(66.0±7.0)/(66.0±8.0)	14/21	15/20	Tid for PreOp 2 days	Tid for PreOp 2 days	Gatifloxacin	Intraoperative mydriasis

RCT = Randomized controlled trial. Cmax = peak aqueous humor concentration. NA = not available.

**Table 2:** 12-item scale critical appraisal scores.

Author	12-item scale critical appraisal score												
	1	2	3	4	5	6	7	8	9	10	11	12	Quatily
**Sahu, S 2015**	Y	Y	N	N	N	Y	Y	Y	Y	Y	Y	Y	**High**
**Duong, HV 2007**	N	Y	Y	Y	Y	Y	Y	Y	Y	Y	Y	Y	**High**
**Malik, A 2016**	Y	Y	N	N	N	Y	Y	Y	Y	Y	Y	Y	**High**
**Ramakrishnan, S 2016**	Y	Y	N	Y	Y	Y	Y	Y	Y	Y	Y	Y	**High**
**Tzelikis, PF 2015**	Y	Y	N	Y	Y	Y	Y	Y	Y	Y	Y	Y	**High**
**Almeida, DR 2012**	N	Y	Y	Y	Y	Y	Y	Y	Y	Y	Y	Y	**High**
**Nardi, M 2007**	N	Y	Y	Y	N	Y	Y	Y	Y	Y	Y	Y	**High**
**Walters, T 2007**	N	Y	Y	Y	N	Y	Y	Y	Y	Y	Y	Y	**High**
**Bucci, FA 2011**	N	Y	Y	Y	Y	Y	Y	Y	Y	Y	Y	Y	**High**
**Bucci, FA 2011**	N	Y	Y	Y	Y	Y	Y	Y	Y	Y	Y	Y	**High**
**Zanetti, FR 2012**	Y	Y	Y	Y	Y	Y	Y	Y	Y	Y	Y	Y	**High**

12-item scale criteria: (1)Method of randomization; (2)Concealed allocation; (3)Patient blinding; (4)Provider blinding; (5)Outcome assessor blinding; (6)Drop-out rate; (7)Patient allocated as plan; (8)Free of selective outcome reporting; (9)Same baseline; (10)Co-interventions avoided or similar; (11)Acceptable compliance; (12)Same time of outcome assessment. Y = Yes, N = No, A trial with a score of 7 or more was considered high quality, more than four but no more than seven was considered moderate quality, and no more than four was considered low quality.

### Best corrected visual acuity

Together, five studies [3, 7, 14–17, 20–24] included 262 patients in Nepafenac group and 274 patients in Ketorolac group described the perioperative BCVA (log MAR scale) respectively.

For preoperative BCVA, a fixed-effect model was used as no heterogeneity was detected (*P* = 0.448, *I*^*2*^ = 0%). The pooling result showed no statistical difference of preoperative BCVA between the two groups (WMD = -0.004, 95%CI: - 0.070~0.063, *P* = 0.909, Table 3).

**Table 3:** Comparison of BCVA between each group at different time periods.

Time period	No. of studies	Sample size		WMD	95% CI		*P* of chi-square	*I*^*2*^	Selected model	*P* for overall effect
		Nepafenac	Ketorolac							
PreOp	3	158	170	-0.004	-0.070	0.063	0.448	0	Fixed-effect model	0.909
PostOp 1 day	2	136	142	0.084	-0.041	0.210	0.808	0	Fixed-effect model	0.188
PostOp 1 week	3	167	175	-0.008	-0.062	0.045	0.154	46.6%	Fixed-effect model	0.754
PostOp 1 month	5	262	274	0.001	-0.024	0.023	0.179	36.4%	Fixed-effect model	0.990

For BCVA at postoperative 1 day, 1 week and 1 month, fixed-effect models were used as no heterogeneity was detected. The forest plots of these 3 time periods also indicated that there was no statistical difference between the Nepafenac group and the Ketorolac group (Table 3).

### Anterior chamber inflammation grade and inflammation free rate

Three studies [3, 17, 20] included 167 patients in the Nepafenac group and 175 patients in the Ketorolac group described the postoperative anterior chamber inflammation grade. When pooling the data of anterior chamber inflammation grade at postoperative 1 day, 1 week and 1 month, fixed-effect models were used as Chi-squared test manifested no heterogeneity. The results of meta-analysis showed no statistical difference of anterior chamber inflammation grade between the two groups at these postoperative periods (Table 4).

**Table 4:** Comparison of anterior chamber inflammation grade between each group at different time periods.

Time period	No. of studies	Sample size		WMD	95% CI		*P* of chi-square	*I*^*2*^	Selected model	*P* for overall effect
		Nepafenac	Ketorolac							
PostOp 1 day	3	167	175	-0.060	-0.255	0.135	0.185	40.7%	Fixed-effect model	0.547
PostOp 1 week	3	167	175	0.025	-0.049	0.099	0.872	0	Fixed-effect model	0.507
PostOp 1 month	3	167	175	0.010	-0.019	0.039	0.755	0	Fixed-effect model	0.508

Two papers [7, 20] reported the inflammation free rate of anterior chamber. Fixed-effect models were used at postoperative 1 day and 1 month; however, a random-effect model was selected as heterogeneity was significant at postoperative 1 week (*P* = 0.078, *I*^2^ = 67.9%). The forest plots of these 3 time periods indicated that the Nepafenac group’s inflammation free rate had no statistical difference with the Ketorolac group (Table 5).

**Table 5:** Comparison of inflammation free rate between each group at different time periods.

Time period	No. of studies	Sample size		RR	95% CI		*P* of chi-square	*I*^*2*^	Selected model	*P* for overall effect
		Nepafenac	Ketorolac							
PostOp 1 day	2	126	123	0.822	0.395	1.712	0.317	0.3%	Fixed-effect model	0.601
PostOp 1 week	2	126	123	0.743	0.356	1.549	0.078	67.9%	Random-effect model	0.428
PostOp 1 month	2	126	123	0.989	0.896	1.092	-	-	-	0.830

### Ocular discomfort and conjunctival hyperemia

**Fig 2:**
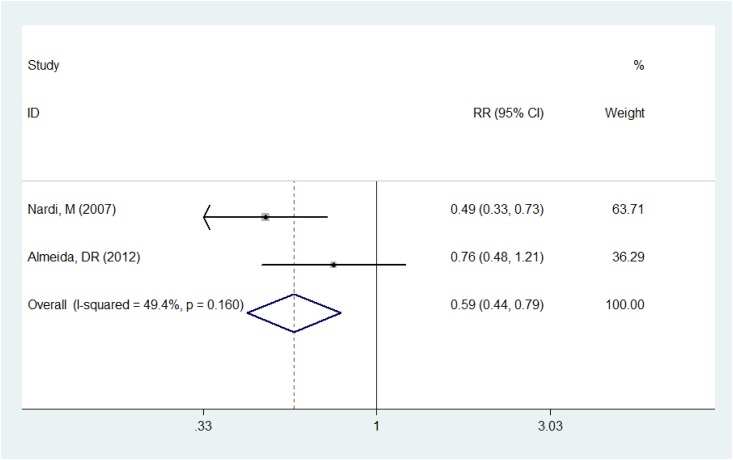
Comparison of postoperative ocular discomfort rate between the Nepafenac group and Ketorolac group.

Two studies [7, 22] described the postoperative ocular discomfort rate, the pooling result by fixed-effect model (*P* = 0.160, *I*^2^ = 49.4%) manifested that the ocular discomfort rate of the Nepafenac group was significantly lower than the Ketorolac group (RR = 0.589, 95%CI:0.436~0.794, *P* = 0.001, Fig 2).

**Fig 3:**
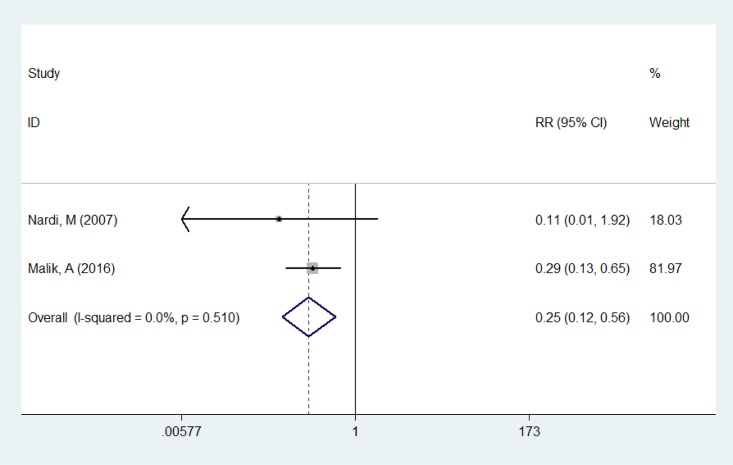
Comparison of postoperative conjunctival hyperemia rate between the Nepafenac group and Ketorolac group.

The rate of postoperative conjunctival hyperemia was calculated in two papers [7, 20], and the fixed-effect model was used as no heterogeneity was detected (*P* = 0.510, *I*^2^ = 0%). The result of meta-analysis indicated that the Nepafenac group’s had a significantly lower rate of postoperative conjunctival hyperemia than the Ketorolac group (RR = 0.253, 95%CI: 0.115~0.557, *P* = 0.001, Fig 3).

### Intraoperative mydriasis

**Fig 4:**
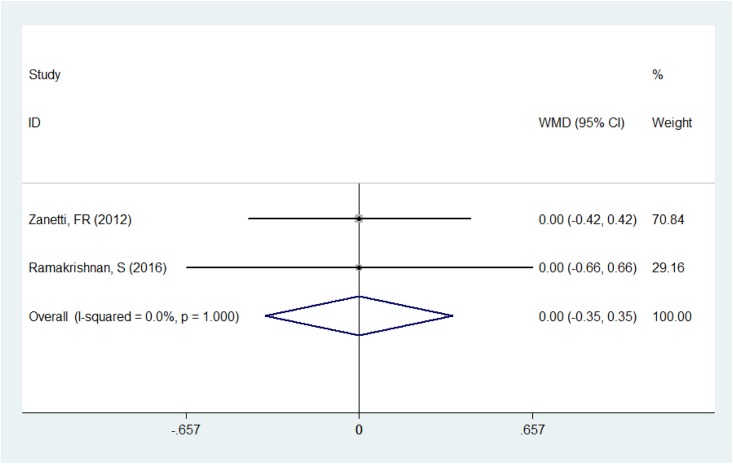
Comparison of intraoperative mydriasis between the Nepafenac group and Ketorolac group.

Two studies [16, 23] described these two drugs’ influence on intraoperative mydriasis. The pooling result of a fixed-effect model (*P* = 1.00, *I*^2^ = 0%) manifested that there was no statistical difference between the Nepafenac and Ketorolac groups in the maintenance of intraoperative mydriasis (WMD = 0.000, 95%CI: - 0.355~0.355, *P* = 1.000, Fig 4).

### Central macular thickness

Two studies [16, 21] included 141 patients in the Nepafenac group and 145 patients in the Ketorolac group described the perioperative CMT. For both preoperative CMT and postoperative CMT at 1 week and 1 month, fixed-effect models were used as no heterogeneity was detected. The forest plots of these 3 time periods manifested that there was no statistical difference between the Nepafenac group and the Ketorolac group (Table 6).

**Table 6:** Comparison of perioperative CMT between the Nepafenac group and Ketorolac group.

Time period	No. of studies	Sample size		WMD	95% CI		*P* of chi-square	*I*^*2*^	Selected model	*P* for overall effect
		Nepafenac	Ketorolac							
PreOp	2	141	145	-0.213	-6.279	5.852	0.896	0	Fixed-effect model	0.945
PostOp 1 week	2	141	145	-1.455	-6.679	3.789	0.577	0	Fixed-effect model	0.588
PostOp 1 month	2	141	145	-2.199	-7.874	3.477	0.771	0	Fixed-effect model	0.448

### Peak drug concentrations and PEG_2_ levels

Two studies [14, 15] calculated the peak aqueous concentrations of Nepafenac, Amfenac (the active metabolite of nepafenac) and Ketorolac. We made the following comparison, since Nepafenac is an inert prodrug and Amfenac is the active metabolite of nepafenac: (1)Amfenac versus Ketorolac; (2) Nepafenac plus Amfenac versus Ketorolac. However, as the heterogeneity of these two comparisons was significantly high (*P* = 0.000, *I*^2^ = 95.7% and *P* = 0.010, *I*^2^ = 85.1%), the pooling results by fixed-effect model and random-effect model were also contradictory, we regarded these pooling results as noneffective.

Bucci et al. [24, 25] performed a serial study and their results indicated that Ketorolac achieved a significantly greater inhibition of PEG_2_ compared to Nepafenac (*P* = 0.025).

### Publication bias

Begg’s test (*P* = 0.462, continuity corrected) and Egger’s test (*P* = 0.680) indicated that publication bias did not affect our results.

## Discussion

Although both Nepafenac and Ketorolac are reported to be effective in the management of postoperative ocular pain and intraocular inflammation after cataract surgery, these two NSAIDs differ structurally and pharmacologically [2]. Ketorolac, which is regarded as benchmark, is not a prodrug and exerts its pharmacological effect by inhibiting prostaglandin biosynthesis after it penetrates the cornea. As a newer topical agent, Nepafenac differs from other NSAIDs because it is administered as a prodrug. Its more neutral and less polarized prodrug structure facilitates its much easier penetration into the cornea and anterior chamber, where conversion to the active form, Amfenac, by intraocular hydrolases happens [7]. The prodrug mechanism of Nepafenac may support the increased activity of Amfenac in the anterior and posterior chamber, with activation in specific areas such as the ciliary body, cornea, iris, retina and choroid. The rapid distribution of Nepafenac may minimize its surface accumulation and associated surface complications that are often observed with other conventional NSAIDs [2]. Theoretically, Nepafenac may have a better efficacy than conventional NSAIDs like Ketorolac both in patients’ tolerability and ocular inflammation associated with cataract surgery. However, several current studies failed to detect the superiority of Nepafenac over Ketorolac and many contradictory conclusions were found both on ocular bioavailability and potency of prostaglandin inhibition [7, 14–17]. Based on the results of our meta-analysis, we evaluated these two drugs for cataract surgery from the aspects of anterior chamber inflammation, visual rehabilitation, patients’ tolerability and intraoperative mydriasis.

We used two representative indices, including anterior chamber inflammation grade and inflammation free rate, to evaluate these two drugs’ efficacy in the management of postoperative ocular inflammation. The pooling results of these two indices indicated no statistical difference between Nepafenac and Ketorolac at postoperative 1 day, 1 week and 1 month, which meant Ketorolac is as effective as Nepafenac in controlling post cataract surgery ocular inflammation. We tried to calculate the peak aqueous concentrations of Nepafenac, Amfenac and Ketorolac [7, 14–17], but we had to regard these comparisons as noneffective beacuse the heterogeneity was significantly high, and the pooling results by fixed-effect model and random-effect model were also contradictory. As for the potency of prostaglandin inhibition, Bucci et al. [24, 25] performed a serial study and their results indicated that Ketorolac achieved a significantly greater inhibition of PEG_2_ compared to Nepafenac; however, we also regarded these results as less reliable, as their data were derived from the same group of subjects. Although some indices are still controversial, we solidly concluded that Ketorolac is as effective as Nepafenac in controlling post cataract surgery ocular inflammation.

Using topical NSAIDs may reduce the risk of developing macular edema after cataract surgery, which means they may lower the risk of poor visual outcome, such as reduced visual acuity and distortion of central vision [26]. In this aspect of visual outcomes, we evaluated the subjective clinical index (BCVA) and objective clinical index (CMT) between Ketorolac and Nepafenac in patients who had cataract surgery. After pooling these data together, we found these two drugs are equally effective in reducing macular edema and achieving a better visual ability. These results are regarded as highly reliable as the heterogeneity was very low, and the comparisons were all done by fixed-effect models.

The forest plots demonstrated that Nepafenac was more effective than Ketorolac in reducing the incidence of postoperative conjunctival hyperemia and ocular discomfort, which were in consistence with the results of previous studies [7, 20, 22]. The reason for this phenomenon could be explained reasonably as the more neutral and less polarized structure of Nepafenac facilitates easier penetration through the cornea. This rapid distribution could minimize its surface accumulation and reduce associated surface complications [2, 7, 10]. Hence, Nepafenac might be more comfortable than Ketorolac in controlling post cataract surgery ocular inflammation.

The miosis that occurs during cataract surgery is partly mediated by prostaglandins. By inhibiting the production of prostaglandins in response to surgical trauma, preoperative treatment using NSAIDs has been reported to be effective in maintaining mydriasis during cataract surgery [2, 23, 27]. The forest plots in our study indicated that there was no significant difference between Ketorolac and Nepafenac in the maintenance of intraoperative mydriasis during cataract surgery, and these two drugs can be applied in surgical practice with similar efficacy.

To our knowledge, this is the first meta-analysis comparing the efficacy and safety between Nepafenac and Ketorolac for prevention and treatment of pain and inflammation following cataract surgery that includes all available evidences in high quality and comprehensively investigates differences in clinical outcomes. The results of our study are highly reliable as the heterogeneity of our analysis is satisfactory and the publication bias is insignificant. Therefore, our study might provide valuable instructions for ophthalmologist. However, it has the following limitations: (1)Although we pooled the data of all the available studies to get the results through the most reliable way, the final sample size was still relatively small, which means more research of high quality should be carried out; (2)The meta-analysis of peak aqueous concentrations of drugs was noneffective and there were insufficient data to analyze the PEG_2_ levels. Further research should focus on these two points as they are the most direct indices for evaluating the efficacy of two drugs.

## Conclusions

Compared with Ketorolac, topical Nepafenac has a superior efficacy in patients’ tolerability following cataract surgery. However, these two drugs are equally desirable in the management of anterior chamber inflammation, visual rehabilitation and intraoperative mydriasis. Given the limitations in our study, further researches with larger sample sizes and focus on more specific indicators such as peak aqueous concentrations of drugs or PEG2 levels are required to reach a firmer conclusion.

## Supporting information

S1 PRISMA Checklist (DOC)
